# Public – private 'partnerships' in health – a global call to action

**DOI:** 10.1186/1478-4505-2-5

**Published:** 2004-07-28

**Authors:** Sania Nishtar

**Affiliations:** 1 Heartfile, Islamabad, Pakistan

**Keywords:** private sector, public sector, partnerships, conflict of interest.

## Abstract

The need for public-private partnerships arose against the backdrop of inadequacies on the part of the public sector to provide public good on their own, in an efficient and effective manner, owing to lack of resources and management issues. These considerations led to the evolution of a range of interface arrangements that brought together organizations with the mandate to offer public good on one hand, and those that could facilitate this goal though the provision of resources, technical expertise or outreach, on the other. The former category includes of governments and intergovernmental agencies and the latter, the *non-profit *and *for-profit *private sector. Though such partnerships create a powerful mechanism for addressing difficult problems by leveraging on the strengths of different partners, they also package complex ethical and process-related challenges. The complex transnational nature of some of these partnership arrangements necessitates that they be guided by a set of global principles and norms. Participation of international agencies warrants that they be set within a comprehensive policy and operational framework within the organizational mandate and involvement of countries requires legislative authorization, within the framework of which, procedural and process related guidelines need to be developed. This paper outlines key ethical and procedural issues inherent to different types of public-private arrangements and issues a *Global Call to Action*.

## Public-private partnerships in health – a global call to action

Public-private partnerships are being increasingly encouraged as part of the comprehensive development framework. The need to foster such arrangements is supported by a clear understanding of the public sectors inability to provide public goods entirely on their own, in an efficient, effective and equitable manner because of lack of resources and management issues. These considerations have necessitated the development of different interface arrangements, which involve the interfacing of organizations that have the mandate to offer public good on one hand, and those that could facilitate this goal.

Within the health sector, public-private partnerships are also the subject of intensely fueled debate [1]. Several examples, which fall within this framework, highlight a potential for the creation of a powerful mechanism for addressing difficult problems by leveraging on the strengths of different partners; however, these also illustrate complex issues, as such arrangements bring together a variety of players with different and sometimes conflicting interests and objectives, working within different governance structures [2].

This paper focuses on public-private partnerships that are intended to address broad questions of providing sustainable health outcomes rather than on the day-to-day interaction that occurs when the government buys a health service from a private supplier or where it leaves the entire matter of health service supply to the private sector.

The *public *sector in this paper refers to national, provincial/state and district governments; municipal administrators, local government institutions, all other government and inter-governmental agencies with the mandate of delivering 'public goods'. The word *private *denotes two sets of structures; the *for-profit *private encompassing commercial enterprises of any size and the *non-profit *private referring to Non Governmental Organizations (NGOs), philanthropies and other not-for-profits. The word *partnership *in this paper refers to long term, task oriented, and formal relationships. There has been ample critique relating to the convention of using the word partnership to describe such arrangements; much of this debate is valid, given that there are certain requisites for coining such an association. For the same reasons it also needs to be differentiated from *privatization*, which involves permanent transfer of control through transfer of ownership right or an arrangement in which the pubic sector shareholder has waived its right to subscribe. A distinction also needs to be made between partnerships and *contractual arrangements*, particularly with regard to the relationship between the public sector and NGOs. Although such arrangements can be used for strategic purposes, they are inherently distinct from *partnerships*.

### Types of public-private interface arrangements

the database of the *Initiative on Public-Private Partnerships for Health of the Global Forum for Health Research *lists 91 international partnership arrangements in the health sector, which can qualify to be called public-private partnerships. Of these, 76 are dedicated to infectious disease prevention and control, notably AIDS, tuberculosis and malaria; four focus on reproductive health issues, three on nutritional deficiencies whereas a minority focus on other issues (health policy and research {1}, injection and chemical safety {2}, digital divide {1}, blindness and cataract {4})[3]. This categorization takes into account large transnational public-private partnerships. There are, however, many other arrangements at a country level, which bring in their wake similar challenges as the ones posed by larger partnerships.

Several classifications have been proposed to conceptualize and categorize public-private partnerships. These may be based on the terms of the constituent membership or the nature of activity [4,5]. However by virtue of the definitions and the characteristics of the *public *and *private *sectors, it can be stated that public-private arrangements are fostered either when governments and inter-governmental agencies interface with the *for-profit *private sector to tap into resources, or the *non-profit *private sector for technical expertise or outreach. Several varieties of arrangements of various sizes, forms and scope at a global, regional or country level qualify to fall within this categorization. Transnational partnerships involving a visible role of the *for-profit *sector are at one end of the spectrum. These usually involve larger partnerships and a complex grouping; depending upon their structure, they may bring together several governments, local and international NGOs, research institutions and UN agencies in transnational programs, often also involving the non-profit sector. Such partnerships can be housed and coordinated by different sources [6]. They can be owned by the pubic sector and have private sector participants such as in the case of Global Alliance for Vaccines and Immunization (GAVI) [7], Roll Back Malaria (RBM) [8], Stop TB partnership (Stop TB) [9], Safe Injections Global Network (SIGN) [10], Global Polio Eradication Programme (PEI) [11], the Special Programme for Research and Training in Tropical Diseases (TDR) [12], and the Special Programme for Research Development and Research Training in Human Reproduction (HRP) [13]. Partnerships can be principally orchestrated by companies such as in the case of Action TB [14], and can be legally independent such as the International Aids Vaccine Initiative (IAVI) [15], Medicines for Malaria Venture (MMV) [16], Global Alliance for TB Drug Development (GATBDD) [17], and the Concept Foundation (CF) [18]. Large partnerships can also be hosted by a civil society NGO; examples include the Malaria Vaccine Initiative (MVI) [19], the Mectizan Donation Programme (MDP) [20], and the HIV Vaccine Initiative (HVI) [21].

At the other end of the spectrum, there are examples of individual governments forming partnerships with the for-profit private sector [22]. There are also examples of situations when a government partners with an NGO with a particular technical strength, technical or outreach related. The recent evolution of a public-private partnership for the prevention and control of non-communicable diseases in Pakistan is an example of this approach, where the government leverages on the technical strength of the private sector partner for addressing an emerging health challenge [23]. Examples also exist of NGOs seeking support from corporate partners both at a national and an international level. The World Heart Federation has recently structured transparent and successful business relationships with the corporate sector for supporting global programs with initial encouraging results [24,25].

Partnerships in the health sector can be for various purposes; categories as stated by the Initiative on Public-Private Partnerships for Health have been summarized in Table 1. Such partnerships are novel arrangements and potentially present an opportunity for more than one partner(s) to contribute to the same goal. Many of these have positively contributed to health outcomes in the past; developing technologies for tropical diseases, surveillance and screening strategies, contributing to technical aspects of sustainable drug development and vector control are amongst a few examples [26,27]. Notwithstanding, partnerships involving the for-profit private sector bring in their wake many concerns as they involve a donor-recipient relationship [28].

**Table 1:** Categorization of public-private partnerships based on the purpose they serve

	Purpose	Partnership
1	Product development	GATBDD, IAVI, MMV and MVI.
2	Improving access to healthcare products	CF, MDP, Accelerated Access Initiative (AAI) [48], Global Alliance to Eliminate Leprosy (GAEL) [49], Global Alliance to Eliminate Lymphatic Filiariasis (GAELF) [50] and the Global Polio Eradication Initiative (GPEI) [51].
3	Global coordination mechanisms	GAVI, RPS, Stop TB, Global Alliance for Improved Nutrition (GAIN) [52], and the Micronutrient Initiative (MI) [53].
4	Strengthening health services	Alliance for Health Policy and Systems Research (AHPSR) [54], Multilateral Initiative on Malaria (MIM) [55], African Comprehensive HIV/AIDS Partnerships (ACHAP) [56].
5	Public advocacy and education	Alliance for Microbicide Development (AMD) [57], African Malaria Partnership (AMP) [58], Global Business Coalition on HIV and AIDS (GBC) [59] and Corporate Council on Africa (CCA) [60].
6	Regulation and quality assurance	The International Conference on Harmonization of Technical Requirements for Registration of Pharmaceuticals for Human Use (ICH) [61], Pharmaceutical Security Institute (PSI) [62] and the Anti-Counterfeit Drug Initiatives [63]

In many countries, there are long established links of the public sector with NGOs. Theoretically, since NGOs are not driven by a profit generating motive, many of the ethical challenges that potentially exist in partnering with the for-profit are not of relevance in this case. However, it could also plausibly be argued that NGOs, who though objective and altruistic, may, in fact, have quite complex motives. In promoting public-private partnerships therefore, several issues need to be clearly flagged in an attempt to address them in tandem with efforts that aim to foster such relationships. Within that context, a set of ethical and process related challenges are summarized hereunder:

#### Ethical challenges, which are largely generic across the range of public-private partnerships relate to the following dimensions

*1. Global norms and principals: *many of the large partnerships involving a variety of players are of a transnational nature. However, against this backdrop, there are no global norms and principals, to set a framework within which global public health goals can be pursued in a partnership arrangement.

*2. Impartiality in health: *if public-private partnerships are not carefully designed, there is a danger that they may reorient the mission of the public sector, interfere with organizational priorities, and weaken their capacity to uphold norms and regulations. Such a shift is likely to displace the focus from the marginalized and may therefore be in conflict with the fundamental concept of equity in health.

*3. Social safety nets: *it has been increasingly argued that engaging in a partnership mode provides the public sector an opportunity to renounce their responsibilities; this in a sense may lead to withdrawal of social safety nets. Failure to commit to maintain the role of the state in such partnerships may result in a *laissez-faire *attitude, prejudicial to the interest of the most vulnerable groups.

*4. Conflict of interest: *many partnerships are initiated on the premise that they fulfill a social obligation, and can involve good intentions on part of individuals and organizations. However the basic motive that drives the 'for-profit' sector *demands *that these involve a financial pay off in the long term. In such cases, the difference between corporate sponsorships and scientific philanthropic donations with long term visible public health goals needs to be clearly separated. This issue has been further complicated in recent years as many global health initiatives funded by endowments generated by foundations have partnerships with the private sector as a key feature [29]. Such donor-recipient relationships bring in their wake many concerns. These include concerns relating to such arrangements providing the 'for profit' private sector an opportunity to improve their organizational image by engaging in cause-related marketing and concerns relating to these engagements facilitating access of the commercial sector to policy makers. On the other hand, many NGOs even in the developing countries are little more than lobby groups with a particular interest, which may or may not be aligned to public good.

*5. Redirecting national health polices: *there are also concerns that such partnerships redirect national and international health polices and priorities and have the potential to defeat crucial local and national efforts.

*6. Fragmentation of the health system: *partnerships generally tend to aim for short term high profile goals and tend to pick the lowest lying fruits. Partnerships do have the mandate and cannot be held accountable to synchronize their activities with emerging processes within countries aimed at developing their health systems. Therefore if they are instituted in countries with weak health systems they have the potential to fragment the healthcare system by instituting independent vertical programs. The changing global agenda around 'vaccines' helps to highlight many of these issues. Previously polices around vaccination were grounded in the general principal that promoted equitable access to few vaccines around the world. However new initiatives and their vertical systems have less of a focus on sustainability, may not contribute to strengthening of the health system and have the potential of redirecting national health policies, which focus on equitable care [30].

*7. Contribution to common goals and objectives: *it is common for partners to have different objectives while pursuing a relationship though it may be implicit that partnerships are contributing to common goals.

*8. Lack of outcome orientation: *many a times, partnerships exist in form and do not contribute to improvements in quality and efficiency.

#### Operational and process-related challenges in public private partnerships relate to the following dimensions

*1. Legislative frameworks, polices and operational strategies: *many developed countries have legislation to interface with the private sector [31]. However in the developing world, there is a general failure, to have overarching legislation relating to public-private partnerships. As a result, such arrangements develop on an *ad hoc *and opportunistic basis and may have questionable credibility; as a results of this failure, polices and specific operational strategies fail to develop.

*2. Participatory approach to decision making: *the expression 'partnership' gives the impression of equality. However many a times, a participatory approach to the decision making process is usually not optimally accomplished. This has implications of varying degrees. Almost all the large 91 transnational partnerships referred to earlier are focused on the developing world. However, among these, 85 have their secretariats in Europe and North America; the United States and Switzerland being the commonest host countries. Cleary this lack of proximity to the intended beneficiaries has a bearing on the manner in which the beneficiaries have a role in the decision making process [32]. The decision-making process in a partnership may also be biased because of the stronger partners' influence. At a county level and in the case of governments interfacing with NGOs, the stronger partner, which his usually the Government generally tends to make the rules. On the other hand, in the case of relationships with the 'for-profit' private sector, there is the danger of the financially stronger partner influencing the public sectors decision making process on policies, regulatory and legislative matters, which have implications for their profit-making motive.

*3. Governance structures: *workable partnerships require a well-defined governance structure to be established to allow for distribution of responsibilities to all the players. Public-private partnerships may run into problems because of ill-defined governance mechanisms. Recent evaluation of the RBM project while acknowledging the successes of the partnership in drawing global attention to the scale of the problem posed by Malaria has outlined serious governance-related issues [33]. More recently, independent evaluation of the Global Stop TB partnership has also resulted in the issuance of detailed recommendations for improved governance [34].

*4. Power Relationships: *skewed power relationships are a major impediment to the development of successful relationships. Governments in developing countries usually tend to assume core responsibility of the joint initiative and take charge of the weaker partner. In case of NGOs with outreach-related strengths, this usually takes the form of a 'contractual relationship' without much regard to the participatory processes, which should be key to a public-private partnership arrangement. In case of relationships with NGOs with technical strength, there are issues relating to power relationships of a more serious nature with regard to who assumes the leadership role.

*9. Criteria for selection: *the criteria for selection are an important issue both from an ethical and process-related perspective as it raises the questions of competence and appropriateness. In many instances the public sector is vague about important issues related to screening potential corporate partners and those in the non-profit sector.

*10. Sustainability: *the question of long-term sustainability is often ignored in public-private partnerships. An analysis of the operation of GAVI has concluded that it overemphasizes high technology vaccines, lacks sustainability, relies too heavily on the private sector and consequently, runs the risk of compounding health inequities in the poorest countries [35].

*11. Accountability: *many partnerships do not ensure that all players are held accountable for the delivery of efficient, effective and equitable services in a partnership arrangement. Often in public-private relationships it is unclear as to whom are these partnerships accountable to, according to what criteria, and who sets priorities? To hold partners accountable for their actions, it is imperative to have clear governance mechanisms and clarify partner's rights and obligations. Clarity in such relationships is needed in order to avoid ambiguities that lead to break up of partnerships. A case in point is the recent breakup of GAEL with the exit of the International Association of Anti-leprosy [36].

## The Call to Action

In the world we live today, global agendas are being increasingly shaped by the private sector. The 'for-profit' private sectors' immense resources make it an irresistible partner for public health initiatives. These arrangements can also be mutually synergistic. Governments and international agencies can tap into additional resources to full fill their mandate whereas the commercial sector can fulfill its social responsibility, for which it is being increasingly challenged. Additionally, the recent SARS epidemic and bio-terrorist threats should help to make the private sector understand the value of investment in health for reasons beyond fulfilling their social obligations. Active involvement of the 'non-profit' sector and donor coordination in country goals is also being increasingly encouraged within comprehensive development frameworks; this approach is synchronous and in harmony with the Poverty Reduction Strategy Paper Framework [37].

The development and health actors have highlighted the need to harness the potential that exists in collaborating with the private sector to advance public health goals. This is also becoming increasingly essential as both the public and the private sector recognize their individual inabilities to address emerging public health issues that continue to be tabled on the international and within country policy agendas. Public-private partnerships therefore seem both, unavoidable and imperative. However in building such collaborations, certain measures must be taken at a global level to assist global partnerships and set a framework within which efforts at a country level can emanate.

As a first step, there is a need to develop a set of global norms and ethical principles; a broad-based agreement over these must be achieved. The transnational nature and global outlook of emerging partnerships necessitate that these stem from a broad-based international dialogue.

It is critical that the driving principles for such initiatives be rooted in 'benefit to the society' rather than 'mutual benefit to the partners' and should center on the concept of equity in health. Norms must stipulate that partnerships contribute to strengthening of social safety nets in disadvantaged settings and should be set within the context of 'social responsibility' as the idea is not meant for private funds to be put to public use nor to privatize public responsibilities.

Global principles must specify that partnerships should be in harmony with national health priorities; they should complement and not duplicate state initiatives and should be optimally integrated with national health systems without any conflict of interest. Norms must make it mandatory for all partners in a 'partnership' arrangement to contribute to common goals as a true partnership is one in which the partners, though having different motivation and values have a shared objective. Global norms must outline that partners be committed to making contributions, sharing risks and the decision making process. Principles should emphasize an outcome orientation. Development of a public-private partnership in itself should not be seen as an *outcome*, but a *process *and an *output*; it is important for partnerships not to just exist in form but to contribute to improvements in health outcomes.

It must be made binding for international agencies to develop transparent policy and procedural frameworks. Many international agencies have established guidelines on interacting with the private sector [38-45]. However there is a need for comprehensive polices and operational strategies, which are crucial to ensuring transparency and protecting public interest [46]. Inviting third party reviews and ensuring an open process for deliberations will help to ensure transparency and reflect that these processes are indeed being structured in public interest.

Global efforts should demand, encourage and assist the development of policy and legislative frameworks shaping public-private partnerships *within *countries [47]. However in the setting of developing countries, there is a need for international actors to guide these and for them to emanate within the framework of global norms and standards. Assisting with capacity development through donor coordination may be a necessary prerequisite to this approach. Legislative and policy frameworks within countries will help to legitimize public-private relationships, lend credence to this approach, help to foster an enabling environment and provide a mandate for the development of ethical guidelines to further direct these initiatives.

Within stipulated legislative and policy frameworks, support must be provided to developing countries to develop specific guidelines to steer such relationships. Guidelines can assist with the development of selection criteria and help specify roles of the public and the private sectors. They can also assist with the development of models that outline combined governance structures, clearly aimed at improved systems of governance. Guidelines must also articulate a clear policy on a participatory approach to the decision making process. In addition, they should assist with assigning responsibilities to various levels of Government and then hold people and institutions both within Governments and those in the private sector that partner with them accountable for their performance.

Though an evidence-based approach and ethical considerations must never be compromised in such endeavors and every effort should be made to ensure that goals are mutually compatible, guidelines also need to be flexible in order to accommodate each partner's organizational requirements and integrity. Moreover they need to be pragmatic. The public sector needs to recognize the basic legitimacy of the private sector and the profit motive that drives it. It is also essential for the public sector to respect the organizational autonomy and priorities of the non-profit sector. In this context, partnerships and contractual relationships need to be carefully differentiated.

Partnerships must also be the subject of noteworthy empirical research, which would enable a detailed assessment of the specific issues inherent to the various types of public-private partnership arrangements from an ethical and methodological perspective.

The impetus for driving global and national efforts in creating a transparent and conducive environment for public-private partnerships needs to come from the public sector. This raises the issue of capacity within countries; the gap needs to be bridged by assistance from UN agencies, which have the mandate of harnessing and coordinating support among a variety of players for global actions. However, the results of such actions will only be as good as Governments make them; weak and poorly informed Governments cannot remedy their own deficiencies by seeking to yolk the private sector to their own uncertain cart.

In conceptualizing a framework that assists with setting global norms and guidelines and within-country legislative actions, it needs to be recognized that the dynamics of public-private partnership arrangements are generic across social sector. It may therefore be useful to allow this commonality to prevail in initiating global and country-specific actions.
